# BRAIN HEALTH COMMUNITY REGISTRY: DEVELOPMENT OF A MODEL FOR CONNECTING PARTICIPANTS TO COMMUNITY-BASED SUPPORTS

**DOI:** 10.1093/geroni/igad104.2467

**Published:** 2023-12-21

**Authors:** Laura Block, Meghan Haas, Lilli Kay, Clark Benson, Andrea Gilmore-Bykovskyi

**Affiliations:** University of Wisconsin-Madison, Madison, Wisconsin, United States; University of Wisconsin-Madison, Madison, Wisconsin, United States; University of Wisconsin-Madison, Madison, Wisconsin, United States; University of Wisconsin School of Medici, Madison, Wisconsin, United States; University of Wisconsin-Madison, Madison, Wisconsin, United States

## Abstract

Growing evidence demonstrates social and economic factors (e.g. socioeconomic status, level of education) contribute to risk for Alzheimer’s disease and related dementia (ADRD). These factors also serve as barriers to research participation, contributing to systematic under-inclusion in ADRD research. The Participant and Relationship-Oriented Research Engagement (PRORE) Model outlines mechanisms through which socioeconomic determinants and reciprocal relationships may shape research participation; however, scalable protocols that address these mechanisms are lacking. Our objective was to develop and pilot the feasibility and acceptability of a research-based resource-matching protocol. The Brain Health Community Registry (BHCR) recruits adults ages 40 and above with and without changes in memory, and caregivers of individuals experiencing memory changes. BHCR connects participants with research opportunities and conducts a needs assessment to match individuals with community-based services and supports. Of 168 participants enrolled in BHCR, 33 (20%) report changes in memory, 93 (55%) do not, and 42 (25%) are caregivers. More than half (n=91, 58%) requested resource matching. Resource matching took 1.8 hours per participant (range 0.5-4.5 hours). Challenges included navigating rural counties with limited resources and effective follow-up; strategies included an interdisciplinary approach, offering multiple modes of contact, and a strengths and relation-based approach. Participants who responded to follow-up at two and six weeks (45% of total), and at annual review (100%, N=6) described resource-matching as instrumental for successful connection to supports, general knowledge/preparation, or on behalf of a friend or family member. Future work must ascertain whether needs assessment and resource matching change study participant perceptions of research.

